# Deaths from Chloroform

**Published:** 1902-01-18

**Authors:** 


### DEATHS FROM CHLOROFORM.

At a recent meeting of the General Medical Council, during a discussion on the teaching of the use of anæsthetics, Dr. Heron Watson is reported to have said that the nurses and dressers were taught the use of anæsthetics in the Scottish hospitals and infirmaries, and that there were no accidents in these institutions with anæsthetics. In the *Lancet* last week a letter appears protesting against these statements, especially against the last. The writer says that accidents do occur there just as in English hospitals; that "in Glasgow during a recent year 50 deaths were registered as due to anæsthetics. In five months 10 occurred in hospitals alone." He argues, we think with justice, that the subject should be more carefully taught than, according to him, is the case.

